# Regulation of IL-17A responses in human airway smooth muscle cells by Oncostatin M

**DOI:** 10.1186/s12931-014-0164-4

**Published:** 2015-02-07

**Authors:** Karen Kwofie, Matthew Scott, Rebecca Rodrigues, Jessica Guerette, Katherine Radford, Parameswaran Nair, Carl D Richards

**Affiliations:** McMaster Immunology Research Centre, Department of Pathology and Molecular Medicine, St Joseph’s Healthcare and McMaster University, Hamilton, Ontario Canada; Division of Respirology, Departments of Medicine, St Joseph’s Healthcare and McMaster University, Hamilton, Ontario Canada; 4017 Michael DeGroote Centre for Learning & Discovery, McMaster University, 1280 Main Street West, Hamilton, ON L8S 4 L8 Canada

**Keywords:** Asthma, Cytokines, Chemokines, STAT signaling, Oncostatin M, Airway smooth muscle

## Abstract

**Background:**

Regulation of human airway smooth muscle cells (HASMC) by cytokines contributes to chemotactic factor levels and thus to inflammatory cell accumulation in lung diseases. Cytokines such as the gp130 family member Oncostatin M (OSM) can act synergistically with Th2 cytokines (IL-4 and IL-13) to modulate lung cells, however whether IL-17A responses by HASMC can be altered is not known.

**Objective:**

To determine the effects of recombinant OSM, or other gp130 cytokines (LIF, IL-31, and IL-6) in regulating HASMC responses to IL-17A, assessing MCP-1/CCL2 and IL-6 expression and cell signaling pathways.

**Methods:**

Cell responses of primary HASMC cultures were measured by the assessment of protein levels in supernatants (ELISA) and mRNA levels (qRT-PCR) in cell extracts. Activation of STAT, MAPK (p38) and Akt pathways were measured by immunoblot. Pharmacological agents were used to assess the effects of inhibition of these pathways.

**Results:**

OSM but not LIF, IL-31 or IL-6 could induce detectable responses in HASMC, elevating MCP-1/CCL2, IL-6 levels and activation of STAT-1, 3, 5, p38 and Akt cell signaling pathways. OSM induced synergistic action with IL-17A enhancing MCP-1/CCL-2 and IL-6 mRNA and protein expression, but not eotaxin-1 expression, while OSM in combination with IL-4 or IL-13 synergistically induced eotaxin-1 and MCP-1/CCL2. OSM elevated steady state mRNA levels of IL-4Rα, OSMRβ and gp130, but not IL-17RA or IL-17RC. Pharmacologic inhibition of STAT3 activation using Stattic down-regulated OSM, OSM/IL-4 or OSM/IL-13, and OSM/IL-17A synergistic responses of MCP-1/CCL-2 induction, whereas, inhibitors of Akt and p38 MAPK resulted in less reduction in MCP-1/CCL2 levels. IL-6 expression was more sensitive to inhibition of p38 (using SB203580) and was affected by Stattic in response to IL-17A/OSM stimulation.

**Conclusions:**

Oncostatin M can regulate HASMC responses alone or in synergy with IL-17A. OSM/IL-17A combinations enhance MCP-1/CCL2 and IL-6 but not eotaxin-1. Thus, OSM through STAT3 activation of HASMC may participate in inflammatory cell recruitment in inflammatory airway disease.

**Electronic supplementary material:**

The online version of this article (doi:10.1186/s12931-014-0164-4) contains supplementary material, which is available to authorized users.

## Background

Allergic asthma involves complex inflammatory pathways that manifest symptoms and pathology that are generally accepted to be influenced by functions of Th2 cells and their products including IL-4, IL-5 and IL-13 [1-3]. These contribute to mechanisms of immune cell accumulation, alterations in airway hyper-responsiveness, excess mucus secretion and increased extracellular deposition around airways [4-7]. Activation of stromal or structural cells such as fibroblasts and airway smooth muscle cells contribute to the process [8,9]. These cells can be regulated by a variety of small molecular weight mediators, growth factors and cytokines to contract and/or to release molecules that further influence inflammation and matrix remodeling. Indeed, the production of cytokines by stromal cells (fibroblasts, epithelial cells and smooth muscle cells) could contribute significantly to the load of a chemokine such as MCP-1/CCL-2. These chemokines can attract monocytes/lymphocytes and fibrocytes [10,11] that potentially generate myofibroblasts and extracellular matrix accumulation. Eotaxin-1 has also been recently shown to stimulate fibrocyte chemotaxis [12] and can be released from human airway smooth muscle cells (HASMC) *in vitro* upon IL-1 or TNF stimulation [13]. HASMC can respond directly to Th2 cytokines [14,15] and with synergy in response to Th2 cytokine and IL-1 combinations [16]. More recently, the role of Th17 cells has become prominent in paradigms of T-helper cell subsets that include Th17, Th1, Th2 and regulatory T cells. IL-17A is the most characterized of the IL-17 family of cytokines (IL-17A through IL-17 F) that also play roles in inflammation, T cell responses and autoimmunity as previously reviewed [17,18]. IL-17A interacts with a receptor complex of IL-17RA/IL-17RC, which is generally expressed on a wide variety of cell types [18]. IL-17A has been detected in asthmatic subjects and been shown to regulate lung fibroblasts [19], epithelial cells[20]and functions of airway smooth muscle cells including chemokine release [21-23],proliferation [24] and contraction [25].

In addition to typical Th2 and Th17 derived cytokines, several sets of studies have implicated the involvement of certain members of the gp130 cytokine family, such as IL-6, and IL-11 (reviewed in [26,27]) in inflammatory airway diseases. The gp130 cytokines include IL-6, IL-11, CT-1, LIF, Oncostatin M (OSM) and IL-31 among others, and are grouped together generally on the basis of their utilization of receptor complexes that require the gp130 signaling chain (with the exception of IL-31). Various family members can function in inflammation, immunity, hematopoiesis, cell differentiation and the regulation of extracellular matrix [28-31]. Among this group, OSM has been demonstrated to regulate stromal cell expression of cytokines and extracellular matrix modulators and have been found to be elevated in chronic conditions such as arthritis [32,33] and psoriasis [34,35]. In addition, evidence indicates elevated levels of OSM in airway inflammation [36-38] and severe asthma [39], where potential roles may involve effects on various structural cells including lung fibroblasts [40], airway epithelial cells [41-43] and airway smooth muscle cells [36,37,44]. Reports have described synergy with OSM /IL-4 combination in inducing eotaxin-1, and OSM/IL-1 combination in inducing VEGF [36,37] expression by HASMC *in vitro*. Here, we assess OSM modulation of IL-17A responses in context of activities of LIF, IL-31, IL-6 and IL-11 *in vitro.* We observe that OSM, but not LIF, IL-31 or other gp130 cytokines, can synergize with IL-17A, IL-4 or IL-13 in chemokine induction, correlating with STAT-3 signaling but not receptor chain alterations. The results indicate that OSM functions in sensitizing HASMC to the presence of Th17 cytokines as well as inflammatory and Th2 cytokines, suggesting an expanded role in exacerbation of airway inflammation in human disease.

## Methods

### Cell cultures and stimulation

Cultures of human airway smooth muscle cells (HASMC) were generated from airways obtained from lung cancer patients (ex–smokers, five diagnosed with COPD and 2 with no other conditions) undergoing thoracic surgery at St Joseph’s Healthcare Hamilton after obtaining their informed consent and with the approval of the local Research Ethics Board (approval RP#00-1839). Smooth muscle cells were isolated from disease-free areas of the airways and expanded in RPMI supplemented with 10% fetal bovine serum (FBS) and 1% L-Glutamine for up to 8 passages as previously described [45]. For analysis of cytokine synthesis, HASMC were plated at a cell density of 10,000- 20,000 cells per well in 24-well or 96-well tissue culture plates and incubated overnight. Cells were washed with phosphate-buffered saline (PBS) and incubated for 3 hours with fresh media containing 2% FBS. Cells were then stimulated with the indicated cytokines in 2% FBS containing media, and 18–24 hour supernatants were later collected and stored for future analysis by ELISA. Recombinant human cytokines, (OSM, LIF, IL-31, IL-11, IL-6, IL-17A, IL-4, IL-13 and IFNγ, azide-free) were purchased from R&D systems (Cedarlane, Burlington, Canada). The STAT-3 inhibitor, Stattic, was purchased from Abcam Biochemical (Toronto, Canada) and used at the indicated concentrations applied for 30 minutes before stimulation with cytokines. The p38 inhibitor (SB203580) and Akt inhibitor (Akt X) were purchased from Calbiochem (San Diego, CA). Cells were pre-treated for 1 hour prior to cytokine stimulation with 10 uM SB203580 or 5 uM Akt X inhibitor.

### Enzyme-linked Immunosorbent Assay (ELISA)

Levels of cytokines in the cell culture supernatants were quantified by ELISA using Duoset antibody pairs for IL-6 and eotaxin-1 purchased from R&D Systems Inc and MCP-1/CCL-2, purchased from Biolegend (San Diego, CA), used as per manufacturer’s protocols. Limit of detection of each ELISA was 15 pg/ml or less.

### RNA extraction and real-time PCR

For RNA analysis, confluent HASMC in T75 culture flasks were washed with phosphate-buffered saline (PBS) and incubated for 3 hours with fresh RPMI media containing 2% FBS. Cells were then washed again and stimulated with fresh medium (2% FBS/RPMI) with the indicated cytokines for 6 or 18 hours. Total RNA was prepared as per manufacturer’s protocols using PureLink RNA Mini kits (Life Technologies, Burlington, Canada), and then analyzed by quantitative real-time PCR (TaqMan) using predetermined assay reagents (PDAR) purchased from Life Technologies (Burlington, Canada) for MCP-1/CCL-2, IL-6, eotaxin-1, eotaxin-3, IL-8, IL-17RA, IL-17RC, IL-4Rα, gp130/IL-6ST and OSMRβ.

### Immunoblots

HASMC were plated at a cell density of 90,000 cells per well in 6-well costars and incubated overnight. Cells were washed with PBS, and incubated for 3 hours in RPMI media containing 2% FBS. Cells were then stimulated in fresh 2% FBS/RPMI containing indicated cytokines for 20 minutes. Cells were then lysed in 200 μL of ice cold RIPA buffer containing protease inhibitors and sheared by passing the cells through a 21-gauge needle 5 times. Protein concentrations of total cell lysates were determined using Bradford assays (Bio-Rad) and denatured by boiling in SDS-containing reducing buffer. 15 μg of total protein were separated by 8% SDS-PAGE and transferred to a nitrocellulose membrane by standard methods. Blots were blocked for 1 hour at room temperature using Licor odyssey blocking buffer (Mandal, Guelph, Canada) and were then probed for the indicated phosphorylated or non-phosphorylated proteins, as indicated in figures, at 4°C overnight. Primary antibodies specific for p-Y-STAT-1, STAT 1, p-Y-STAT3, STAT3, p-Y STAT5, STAT5, p-Y-STAT6, pT/Y-p38, p38, p-T/Y-JNK, p-S-Akt, and Akt were purchased from Cell Signaling Technology (New England Biolabs Ltd., Canada). Primary antibodies specific for Actin and STAT6 were purchased from Santa Cruz Biotechnology (Santa Cruz, CA). Primary antibodies were diluted 1:1000 in odyssey buffer and were detected using Licor anti-Rabbit or anti-Goat IRDye infrared secondary antibodies at 1:5000 dilution (Mandal), and imaged using a Licor odyssey infrared scanner (Mandal).

### Statistical analysis

GraphPad Prism version 5.0 for Macs (GraphPad Software, San Diego, CA) was used for generating graphs and statistical analyses. Figures represent mean values ± standard deviation (SD). The student’s one-tailed *t* test, One- or Two-Way analysis of variance (ANOVA) with Tukey or Bonferroni post-tests were used to assess statistical differences between means; where values of *p < 0.05* were considered statistically significant.

## Results

### Regulation of HASMC by OSM and IL-17A

**Figure 1:**
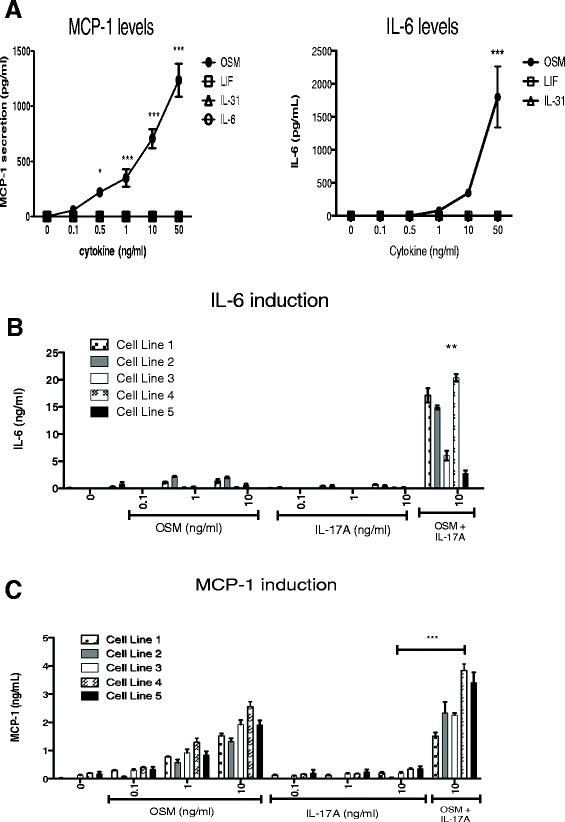
**HASMC responses to OSM and IL-17A.** HASMC were cultured and prepared for stimulation as described in methods, where each treatment is completed in quadruplicates within each cell line. Cells were then stimulated in medium supplemented with 2% FBS and 0–50 ng/ml of the indicated gp130 cytokines. Protein concentrations in 24-hour supernatants were determined by ELISA for **(A)** MCP-1/CCL-2 (Left panel) and IL-6 (Right panel). *p < 0.05; ***p < 0.001 indicates statistical significance compared to no treatment as well as other indicated cytokines using One-Way ANOVA with Bonferroni’s post-test. Panel **(B)** (IL-6) and **(C)** (MCP-1) shows 24-hour supernatants from independent cell lines derived from 5 patients, prepared as above and stimulated with the indicated amounts of OSM, IL-17A or the combination as indicated. **p < 0.01 comparing indicated cytokines combinations to either cytokine alone using One-Way ANOVA with Bonferroni’s post-test.

**Figure 2:**
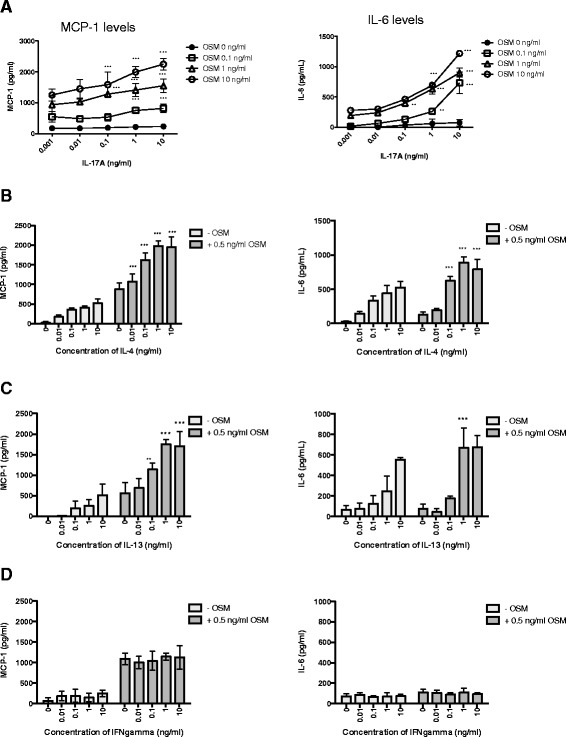
**OSM stimulation with IL-17A, IL-4 or IL-13 induces MCP-1/CCL2 and IL-6.** HASMC cultures were prepared and stimulated as in Figure 1 with the indicated cytokines (in quadruplicates) and concentrations of: **(A)** IL-17A up to 10 ng/ml and in combination with 0, 0.01, 0.1, 1, and 10 ng/ml OSM, **(B)** IL-4 at 0 to 10 ng/ml and in combination with 0 or 0.5 ng/ml OSM, **(C)** IL-13 at 0 to 10 ng/ml and in combination with 0 or 0.5 ng/ml OSM, **(D)** IFNγ at 0 to 10 ng/ml and in combination with 0 or 0.5 ng/ml OSM. Data shown are from one of two individual cell lines that showed identical trends. 24-hour supernatants were collected and cytokine concentrations were quantified by ELISA for MCP-1/CCL-2 (left panels) or IL-6 (right panels). *p < 0.05; **p < 0.01; ***p < 0.001 comparing indicated cytokine combinations to either cytokine alone using Two-Way ANOVA with Bonferroni’s post-test.

We first examined the response of HASMC to recombinant OSM in the regulation of MCP-1/CCL-2 and IL-6 using ELISA systems for protein analysis. We observed a dose-responsive induction of MCP-1/CCL-2 (Figure 1A, left panel), detectable at 0.5 ng/ml and further elevated at higher concentrations of OSM. When stimulating HASMC cultures with IL-6, LIF or IL-31, at concentrations ranging up to 50 ng/ml, we did not observe detectable changes in MCP-1/CCL-2 supernatant levels. OSM stimulation also elevated IL-6 in supernatants of HASMC (Figure 1A, right panel), while we did not detect any changes in IL-6 upon stimulation with LIF or IL-31 in this system. To assess the effects of IL-17A in regulation of HASMC, we cultured cells with increasing doses of IL-17A (Figure 1B and C). We observed minimal responses to doses of IL-17A tested alone (0.1 to 10 ng/ml) when measuring IL-6 or MCP-1 levels in supernatants. However, we were able to detect synergistic induction of IL-6 (Figure 1B) and MCP-1 (Figure 1C) levels in supernatants upon co-stimulation with 10 ng/ml OSM and IL-17A. This was consistently evident in different HASMC cell lines examined, albeit the magnitude of responses varied somewhat between cell lines. Baseline levels of MCP-1 (approximate mean of 170 ug/ml, Figure 1C and 175 ug/ml in Figure 2A) were not statistically altered by stimulation with 10 ng/ml IL-17A (approximate mean of 225 ug/ml in Figure 1C and 235 ug/ml in Figure 2A). To determine whether lower concentrations of OSM could induce synergy with IL-17A, we cultured cells with increasing doses of IL-17A in the presence of 0, 0.1, 1 or 10 ng/ml OSM. Levels of MCP-1/CCL-2 (Figure 2A, left panel) and IL-6 (Figure 2A, right panel) showed that addition of OSM at 1 or 10 ng/ml could induce significant increases in MCP-1/CCL-2 or IL-6 responses to IL-17A.

We next assessed whether OSM could synergize with typical Th1 (IFNγ) or Th2 (IL-4, IL-13) cytokines in the regulation of MCP-1/CCL-2 by HASMC. Figure 2B and C show that OSM (0.5 ng/ml) co-stimulation with IL-4 or IL-13 resulted in synergistic increases in MCP-1/CCL-2 (left panels) and IL-6 levels were also elevated upon co-stimulation (right panels). On the other hand, OSM and IFNγ co-stimulations did not result in MCP-1/CCL-2 or IL-6 modulation differently than either cytokine alone (Figure 2D left and right panels). In the assessment of eotaxin-3 mRNA (Additional file 1: Figure S2), the function of OSM or IL-17A was negligible in comparison to that of IL-4 or IL-13. This is in contrast to IL-8 responses, which showed marked up-regulation by OSM/IL-17A combinations and not by IL-4/OSM or IL-13/OSM (Additional file 1: Figure S3).

### Regulation of HASMC by other gp130 cytokines

**Figure 3:**
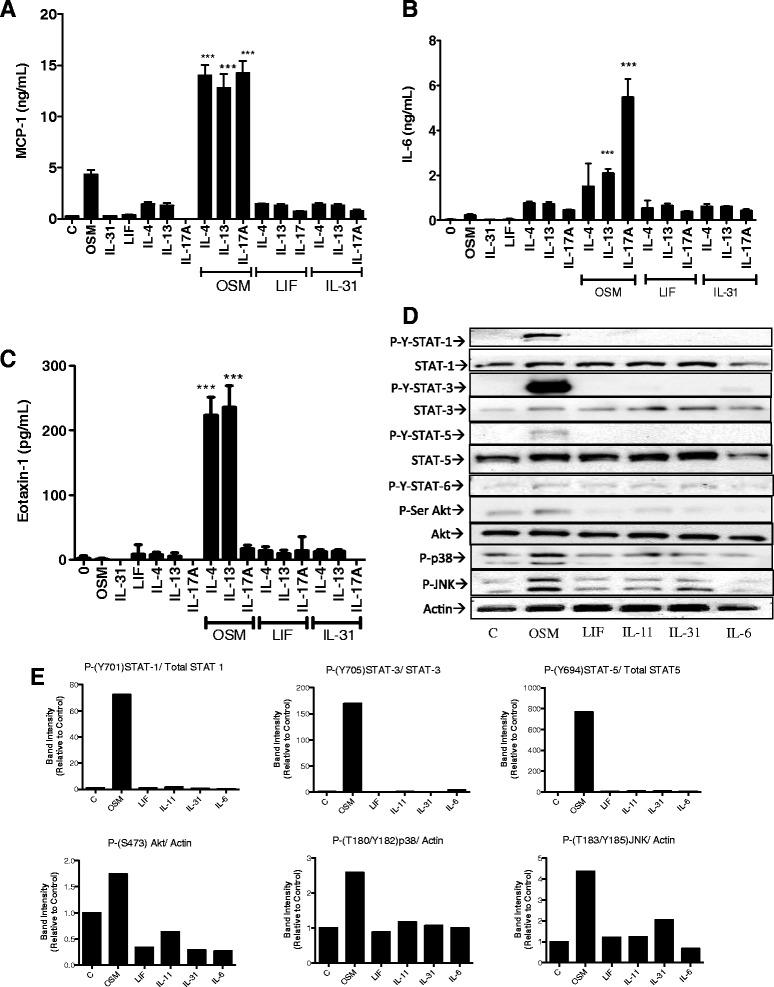
**OSM and other gp130 cytokine stimulation and HASMC responses. (A-C)** HASMC cultures were prepared and stimulated as in Figure 1 with the indicated cytokines and concentrations of 10 ng/ml for each. Data shown are from one of two individual cell lines that showed identical trends. 24-hour culture supernatants were assessed by ELISA for levels of MCP-1/CCL-2 (A), IL-6 **(B)** and eotaxin-1 **(C)**. ***p < 0.001 indicates statistical significance comparing indicated cytokine combinations to either cytokine alone using One-Way ANOVA with Bonferroni’s post-test. **(D)** HASMC cultures were stimulated with OSM, LIF, IL-11, IL-31 or IL-6 (10 ng/ml) for 20 minutes and cell lysates were prepared for immunoblots as described in methods. The lysates were probed for p-Y-STAT1, STAT1, p-Y-STAT3, STAT3, p-Y- STAT5, STAT5, p-Y-STAT6, p-Ser-Akt, p-T/Y-p38, p-T/Y-JNK and Actin as indicated. **(E)** Quantitative analysis of band intensity using densitometry (ImageJ) corrected to Actin or total protein for each probe and expressed as fold change relative to control (unstimulated).

The gp130 cytokine family shares several biological functions, mediated through gp130 signaling, in cells that express the specific ligand receptor chains. We thus assessed whether LIF (utilizes the complex of gp130 and LIFRα), IL-31 (utilizes the complex of IL-31R and OSMRβ chain), or other members of the gp130 cytokine family could regulate HASMC expression of MCP-1/CCL-2 in context of IL-4, IL-13 or IL-17A co-stimulation (Figure 3). MCP-1/CCL-2 levels (Figure 3A) were synergistically induced by the combination of OSM with IL-17A, IL-4 or IL-13. IL-6 levels (Figure 3B) were most pronounced with OSM and IL-17A combinations. Eotaxin-1 levels were induced synergistically by OSM in combination with IL-4 or IL-13, but not in combination with IL-17A (Figure 3C). Parallel testing of IL-31 or LIF (used at 10 ng/ml) shown in Figure 3A-C, showed no effects in the modulation of MCP-1/CCL-2, IL-6, or eotaxin-1 in this system alone or in combination with IL-17A, nor did IL-11 or IL-6 (shown in Additional file 1: Figure S1). In testing the effects of stimulation in serum-free conditions (2 days quiesed in RPMI/1%BSA before stimulation), the response to OSM or IL-17A, and the OSM/IL-17A combination, was identical in trend in the resulting levels of MCP-1and IL-6 found in the cell supernatants (Additional file 1: Figure S4 A, B), although we observed lower absolute quantities than the 2% FBS/RPMI conditions. We also observed identical trends with IL-4, OSM and IL-4/OSM regulation of eotaxin-1 in the same serum-free culture conditions (Additional file 1: Figure S4C) compared to 2% FBS conditions.

Since activation of the JAK-STAT signaling cascade is a prominent intracellular signaling pathway in response to gp130 cytokines, we assessed levels of tyr-phosphorylated STAT1, STAT3, STAT5 and STAT6 in HASMC lysates after cytokine stimulation (Figure 3D) as well as MAPK (p38 and JNK) and Akt pathway activation by standard immunoblots (Figure 3D). Quantitative assessment of band intensities through densitometry (Figure 3E) show that signals for P-STAT-1, 3 and 5 were upregulated by OSM but not by the other members of the gp130 cytokines tested in these cells. Signals for p-(T/Y)-JNK, p-(T/Y)-p38 and p-(S)-Akt were also upregulated by OSM, but minimal or no signals were observed upon stimulation with the other gp130 cytokines tested in these cells. This was consistent in two separate cell lines examined. We also observed that the regulation of STAT3 and p38 cell signaling (phosphorylation) in serum-free conditions were also identical in trend (Additional file 1: Figure S5) to that in 2% FBS/RPMI conditions. OSM-induced signals were evident and other gp130 cytokine signals were low/absent, although again, the levels of signals were much lower in serum free (RPMI/ 1% BSA) conditions.

### Regulation of mRNA levels by OSM in HASMC

**Figure 4:**
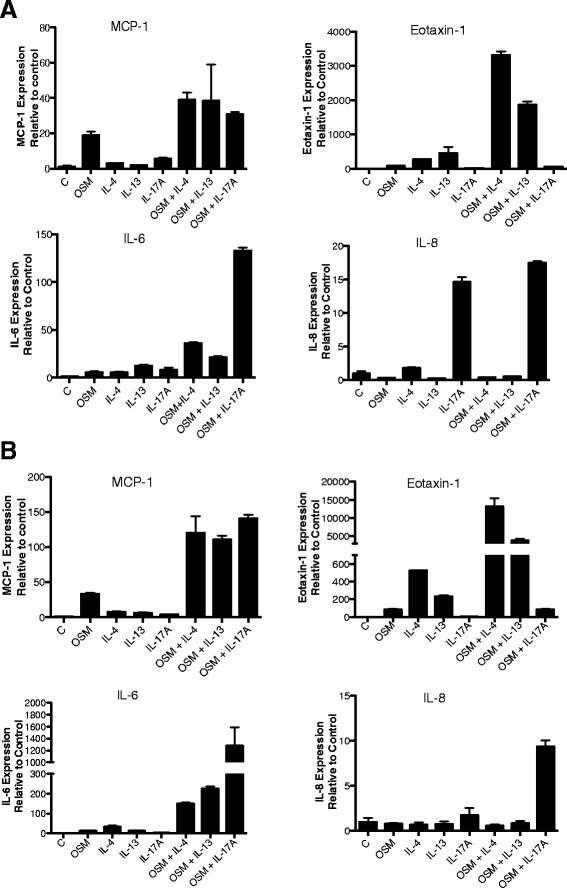
**Regulation of chemokine RNA in HASMC by cytokines.** HASMC were plated and prepared for RNA analysis as in methods after 6 hours **(A)** and 18 hours **(B)** of stimulation with OSM at 2 ng/ml and/or the indicated cytokines at 5 ng/ml. RNA was prepared and analyzed by qRT-PCR using probes for the cytokines as indicated in methods. Levels are expressed as fold change relative to unstimulated (control) and corrected to β-Actin as an endogenous reference control.

To assess whether the regulation of the protein was linked to mRNA regulation, we measured mRNA levels in HASMC after stimulation with OSM, IL-4, IL-13 and IL-17A at both 6 hours (Figure 4A) and at 18 hours (Figure 4B). Changes in levels of mRNA reflected trends seen in the protein levels. OSM and IL-17A co-stimulation synergistically regulated MCP-1, IL-6 and IL-8, but not eotaxin-1 mRNA (Figure 4). OSM with either IL-4 or IL-13 synergized to induce MCP-1, IL-6 and eotaxin-1 but not IL-8 mRNA (Figure 4). Although eotaxin-3 mRNA was regulated by IL-4 or IL-13 (Additional file 1: Figure S2), we did not observe eotaxin-3 regulation by IL-17A, OSM, or the combinations. Furthermore, we were not able to detect eotaxin-3 in HASMC culture supernatants by ELISA (data not shown).

**Figure 5:**
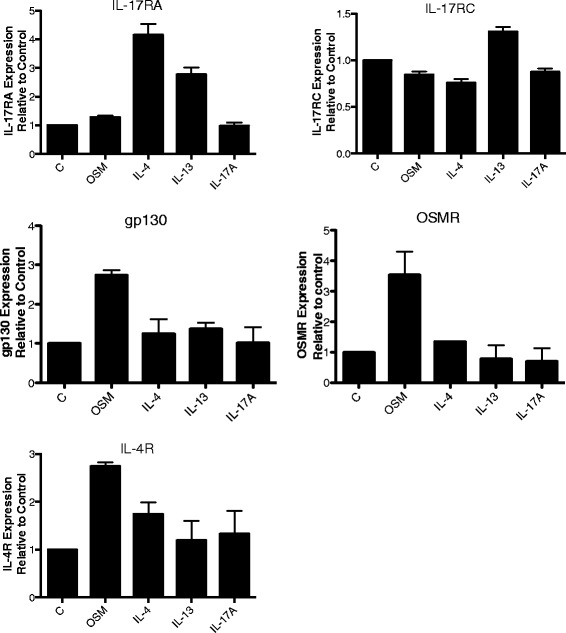
**Receptor chain mRNA levels in stimulated HASMC.** HASMC were plated and prepared for RNA analysis as described in methods after 6 hours of stimulation with the indicated cytokines at 5 ng/ml or 2 ng/ml OSM (n = 2). RNA was prepared and analyzed by qRT-PCR using probes for the receptor subunits IL-17RA, IL-17RC, OSMRβ, gp130/IL-6ST and IL-4Rα, as indicated. Levels are expressed as fold change relative to unstimulated (control) and corrected to β-Actin as an endogenous reference control*.*

Since the regulation of receptor expression may influence responses by HASMC, we assessed the mRNA expression of receptor chains IL-17RA, IL-17RC, gp130, OSMRβ and IL-4Rα (Figure 5). OSM did not alter levels of IL-17RA or IL-17RC mRNA at 6 hours. Both IL-4 and IL-13 enhanced IL-17RA levels (over 4-fold and 2-fold, respectively) but did not regulate OSMRβ mRNA. OSM stimulation for 6 hours resulted in induction of mRNA levels for IL-4Rα 2.5-3 fold, and its own receptor chains OSMRβ and gp130 (approximately 4 fold and 2.3-fold, respectively). These altered levels showed the same trend at 18 hours (not shown). IL-17A stimulation did not regulate IL-4Rα, IL-17RA, IL-17RC, gp130 or OSMRβ, in context of its marked elevation of IL-8 mRNA levels (Figure 4).

### Activation of cell signaling and effects of inhibitors

**Figure 6:**
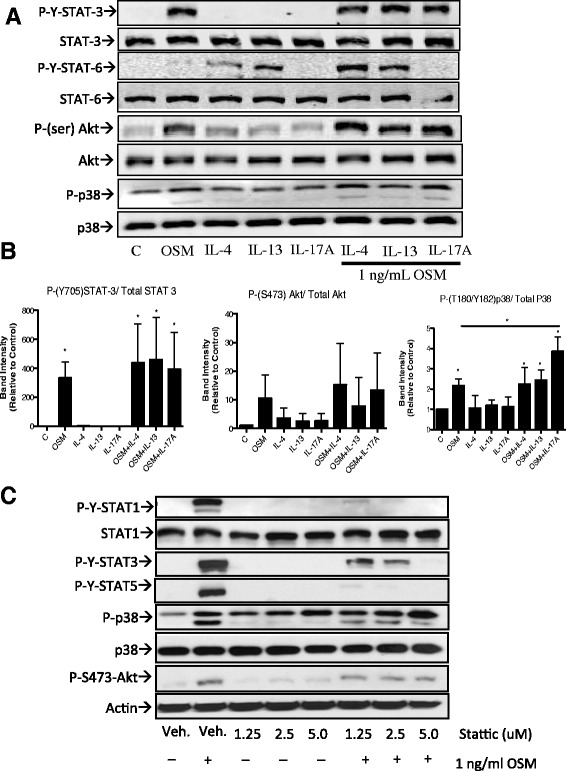
**STAT3, p38 (MAPK) and Akt activation in HASMC and effects of inhibition on HASMC responses. (A)** HASMC cultures were prepared as previously outlined for immunoblots of lysates of cells stimulated for 20 minutes with 5 ng/ml of IL-4, IL-13 and IL-17A (and/or 1 ng/ml of OSM). Total cell lysates from 3 different cell lines were probed for p-Y-STAT3, STAT3, p-Y-STAT6, STAT6, p-S-Akt, Akt, p-T/Y-p38, and total p38 as indicated (one representative cell line shown). **(B)** Quantification of band intensity using densitometry (ImageJ) corrected to total protein for each probe and expressed as the average (3 cell lines) fold change relative to control (un-stimulated) *p <0.05 using one-tail *t-*test. **(C)**. HASMC cultures were prepared as previous for immunoblots of lysates of cells stimulated for 20 minutes with 1 ng/ml OSM, with or without pre-incubation with 1.25, or 2.5 or 5 uM Stattic or its vehicle DMSO (Veh). The lysates were probed for p-Y-STAT1, total STAT1, p-Y-STAT3, p-Y-STAT5, p-T/Y-p38, total p38, p-S-Akt and actin as indicated.

**Figure 7:**
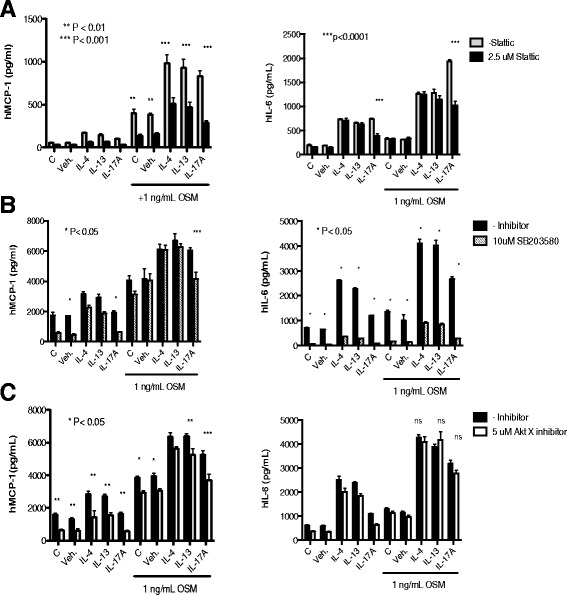
**Effects of STAT3 pharmacologic inhibition on HASMC responses.** HASMC cultures were prepared and stimulated, as previous, with the indicated cytokines IL-4, IL-13, or IL-17A at 5 ng/ml in the absence or presence of 1 ng/ml OSM. Parallel cultures were stimulated in the presence of either **(A)** 2.5 uM Stattic (black bars), **(B)** 10 uM SB203580 (dashed lines) or **(C)** 5 uM Akt X (white bars). Cell lines were stimulated for 18 hours and cell culture supernatants were collected and assessed by ELISA for levels of MCP-1/CCL-2 (left panel) and IL-6 (right panels). *p < 0.05; **p < 0.01, ***p < 0.001 comparing presence and absence of the indicated inhibitor in each cytokine treatment with 1 ng/ml OSM using Two-Way ANOVA with Bonferroni’s post-test.

To determine if selective signaling pathways were amplified with the combinations of cytokines, we assessed phosphorylation of STAT3, Akt at serine 473 and p38 by immunoblots (Figure 6A) and quantitative densitometry for 3 different HASMC cell lines (Figure 6B). p-(Y)-STAT3, p-(S)-Akt and p-(T/Y)-p38 levels were up-regulated by OSM alone and in combination with the other stimuli but not by IL-4, IL-13 and IL-17A alone in this system (Figure 6A). Levels of STAT3 or Akt activation were not statistically different in combination stimuli verses OSM alone in averaging the 3 cell line responses (Figure 6B, left and middle panels). However, levels of p38 phosphorylation on average were further increased (approx.1.7-fold, statistically significant) with OSM/IL-17A combination stimulations of HASMC (Figure 6B right panel). To examine whether inhibition of these pathways could alter HASMC responses to OSM and the synergistic responses induced by OSM combined stimuli, we assessed HASMC responses in the presence or absence of pharmacological inhibitors of STAT-3 (Stattic), p38 (SB203580) and Akt (Akt X). To test the effective concentrations and specificity of Stattic on STAT inhibition, 1.25, 2.5 or 5 uM Stattic was used to pre-treat HASMC cell cultures before stimulating with OSM (1 ng/ml). Levels of phosphorylated STAT1, 3, 5, p38 and Akt were assessed by immunoblots (Figure 6C). p-(Y)-STAT-1,3,5 signals at 20 minutes OSM stimulation were inhibited by 1.25 uM, more so by 2.5 uM and nearly complete reduction at 5 uM Stattic pre-incubation. Stattic is described as a STAT3 selective inhibitor [46,47] however; in our system Stattic markedly inhibited STAT-1 and STAT-5 activation as well. When we examined the effects of Stattic on other cell signaling pathways MAPK (p38) and Akt, we observed minimal reduction in p38 and Akt phosphorylation and a lack of dose-dependent effects (Figure 6C). To assess Stattic effects on MCP-1/CCL-2 or IL-6 production, 2.5 uM Stattic was used to pre-treat HASMC cultures before stimulation with the cytokines and combinations indicated (Figure 7A). Cell viability was not altered at this concentration of the inhibitor. Elevation of MCP-1/CCL-2 levels in response to OSM, or the synergistic MCP-1/CCL-2 responses to OSM/IL-4, OSM/IL-13 or OSM/IL-17A were reduced by 50% or more at 1.25 uM Stattic (not shown) and by 60% or greater at 2.5 uM Stattic (Figure 7A left panel). In contrast, Stattic did not alter IL-6 levels in response to the various stimuli, with the exception of the OSM/IL-17A combination (Figure 7A, right panel). This suggests that STAT-3 has a selective role in OSM/IL-17A synergy.

We next examined whether inhibition of p38 (Figure 7B) and Akt (Figure 7C) would alter cytokine responses in HASMC. MCP-1/CCL2 levels induced by IL-17A alone or in combination with OSM were significantly inhibited by SB203580 at the concentration used but were not significantly reduced in the other combinations (Figure 7B, left panel). IL-6 levels (Figure 7B right panel) were significantly inhibited by SB203580 regardless of the stimulation used in HASMC cultures. The Akt X inhibitor reduced MCP-1/CCL2 levels in supernatants of cultured HASMC independent of the stimuli used (Figure 7C, left panel) but did not inhibit IL-6 levels (Figure 7C, right).

## Discussion

Collectively, our results indicate that OSM has a unique function in HASMC by stimulating synergistic responses to IL-17A in selective genes, (MCP-1/CCL-2, IL-6 and IL-8 but not eotaxin-1). This is supported by data assessing both the protein released on cell culture supernatants (Figures 1, 2 and 3) and mRNA analysis (Figure 4). In addition, the synergistic responses by OSM were not associated with regulation of the receptor chains for IL-17A (IL-17RA and IL-17RC), but were correlated with the level of STAT-3 and p38 activation (Figure 3 and Figure 7). Furthermore, related gp130 cytokine family members LIF, IL-6, or IL-31 could not regulate such synergistic responses (Figure 3, Additional file 1: Figure S1), indicating novel activities of OSM in the regulation of chemokine expression by HASMC *in vitro*. OSM could dose-dependently lower the threshold to IL-17A responses with OSM concentrations as low as 0.1 ng/ml and was clearly evident at 1 ng/ml (Figures 1 and 2). These concentrations are within the range of those detected in sputum samples of patients with severe asthma as described by Simpson et al. [39] and indicate physiological relevance of the observations suggesting OSM regulation of HASMC function in lung inflammation *in vivo*.

Previous work examining HASMC responses to IL-17A showed induction of IL-8 (CXCL-8) [21] and induction of eotaxin-1/CCL-11 [22] through a STAT3-dependent pathway [48]. Our results are consistent with the regulation of IL-8 by IL-17A alone, and show additional synergistic elevation of IL-8 at both the mRNA and protein levels upon OSM/IL-17A co-stimulation. We also observed markedly high levels of eotaxin-1/CCL-11 or MCP-1/CCL-2 with OSM/IL-4 or OSM/IL-13 stimulation (Figures 2 and 3) consistent with previous work by others [36]. In contrast to previous studies on eotaxin-1/CCL-11 expression [22,48], which were completed in serum-free conditions, in our study here (culture conditions of 2% FBS/RPMI) we found minimal regulation of eotaxin-1 using IL-17A alone or in combination with OSM. Figure 6A and B shows the analysis with densitometry averaged from 3 separate cell lines, all showing a lack of IL-17A induction of either STAT3 or p38 in our system. To address this, we compared 2% FBS/RPMI to serum-free conditions but observed the same trend in our system (two different cell lines tested, Additional file 1: Figures 4C and 6C). Furthermore, IL-17A/OSM-induced responses were clearly evident for IL-8, MCP-1 and IL-6 in the same supernatants assessed for eotaxin-1, and in the same samples assessed at the mRNA level. It is not clear why our observations are not consistent with these previous works on IL-17A (alone) induction of STAT3 or eotaxin-1 but may relate to phenotypic differences in the cell cultures selected by chance for our study in context of the IL-17A concentrations we used. Collectively, we suggest that the comparative role of IL-17A in eotaxin-1 expression by HASMC is less than that of IL-4 or IL-13 in the presence of OSM.

The gp130 cytokine family members have overlapping functions in various cells however it is also clear that unique functions can be ascribed to individual gp130 cytokines on the basis of differential cell signaling pathway engagement and/or differences in cell/tissue receptor chain expression [41,49]. In human systems, OSM can bind and signal through either the specific OSMR complex (OSMRβ chain and gp130 chain, termed OSMR Type II) or the LIFR complex (LIFRα and gp130, also termed OSMR Type I). The OSM receptor complex utilizes the OSMRβ chain that is also a necessary chain of the IL-31 receptor complex [31].

Since LIF did not regulate detectable HASMC cytokine release, and low/absent STAT-3 activation, we conclude that LIFR complex (or OSMR Type I) is minimally functional in HASMC (likely due to low expression of LIFRα subunit) and that OSM functions in HASMC are through the specific complex OSMR Type II. IL-31 appears to have a suppressive role in models of Th2-induced lung inflammation [50] although evidence also suggests it can induce dermatitis in animal models [51]. In our system here, IL-31 did not induce HASMC responses, suggesting its roles may not include modulation of airway smooth muscle cells.

Previous reports have shown elevation of IL-4Rα expression in HASMC by OSM stimulation and our results confirm this at the mRNA level, however OSM did not induce detectable alterations in the IL-17RA or IL-17RC receptor chain mRNA (Figure 5). Collectively, these observations suggest that OSM/IL-17A synergistic responses may not involve receptor regulation, unlike that of OSM/IL-4 or OSM/IL-13 responses. The regulation of OSM/IL-17A responses appears to involve multiple signaling pathways, where pharmacological STAT-3 or p38 inhibition modulated MCP-1/CCL-2 and IL-6 responses. p38 inhibition selectively modulated MCP-1/CCL-2 responses to OSM/IL-17A stimulation and not OSM/IL-4 or OSM/IL-13 stimulation of MCP-1/CCL-2 in this system (Figure 7). The results suggest that targeting p38 MAPK may reduce IL-6 but not other important chemokines such as MCP-1/CCL2, and that targeting STAT signaling may reduce MCP-1/CCL2 in airway smooth muscle in asthma.

The OSM-induced synergy with IL-17A will likely add to the mediation of cell responses in mixed cytokine milieus in inflammatory conditions. OSM can synergize with IL-4/IL-13 as previously shown by others [36] who have also shown OSM/IL-1 synergy [37]. HASMC respond directly to Th2 cytokines [14] and Okada et al. [52] suggests differential regulation of eotaxin-1 by Th1 and Th2 cytokines. In our study here, we did not observe activity of IFNγ nor of OSM/IFNγ combinations (Figure 2). Thus, we speculate that OSM selectively accentuates Th2 and Th17 cytokine responses in HASMC by decreasing the concentrations of such cytokines required to activate these cells. This would enable higher responses to low levels of IL-17A, IL-4 and IL-13 and thus contribute to more marked inflammatory effects such as those seen in severe asthma.

## Summary and conclusion

The regulation of cytokine/chemokine expression in HASMC may contribute significantly to mechanisms of pathology in asthma. MCP-1/CCL-2 interacts with CCR-2 and has chemoattractant activity for CCR-2 positive cells such as monocytes/T cells and in addition is chemotactic and an activator for fibrocytes in mouse [53] or human [11] systems. Fibrocytes, a circulating population of CD45+ coll1+ cells that accumulate at sites of inflammation, are thought to contribute to the increased extracellular matrix in inflammatory lung conditions including asthma [10,54,55]. OSM can enhance IL-4, IL-13 or IL-17A induction of MCP-1/CCL-2, suggesting multiple ways by which OSM may contribute to monocyte and fibrocyte involvement in increased fibrosis seen in severe asthmatic patients. Taken together, the synergy of OSM with IL-4/IL-13 or IL-17A on cytokine and chemokine production by HASMC suggests a potential role for OSM in perpetuating airway inflammation and remodeling. OSM activity will likely include other cell types *in vivo* such as lung fibroblasts, which respond to OSM through STAT3 mediation with functions that promote tissue remodeling [56] .The observations that OSM can sensitize HASMC responses to the presence of lower concentrations of several cytokines implicated in asthma suggest a significant contribution to the severity of exacerbations and inflammatory pathology. It may be particularly relevant in patients with severe asthma who have incomplete bronchodilator reversibility [35] given its role in modulating airway smooth muscle biology.

## Additional file

Additional file 1: Figure S1. HASMC responses to gp130 cytokines. HASMC cultures were stimulated with 10ng/ml of LIF, IL-6, IL-31 or IL-11 and with IL-17A (0–10 ng/ml). 24-hour supernatant levels were quantified by ELISA for MCP-1/CCL-2 (upper panel) or IL-6 (lower panel). **Figure S2.** mRNA levels of Eotaxin-3. HASMC cultures were assessed for eotaxin-3 RNA (qRT-PCR) after 6 hours (top) and 18 hours (bottom) of stimulation with the indicated cytokines at 5ng/ml (OSM at 2 ng/ml). Fold change from control, corrected to β-Actin, is shown. **Figure S3.** IL-8 protein regulation by OSM/IL-17A. HASMC cultures were stimulated with 5 ng/ml of IL-4, IL-13 or IL-17A and OSM (1 or 5 ng/ml). 18-hour supernatant levels were quantified by ELISA. ****P<0.0001* (One-Way ANOVA) compared to control or OSM treatment alone. **Figure S4.** OSM stimulation in serum free conditions. HASMC cultures were stimulated in serum-free RPMI/1% BSA. 24-hour supernatant levels were quantified by ELISA for (A) MCP-1 and (B) IL-6. Panel C: HASMC cultures were stimulated with 10 ng/ml of IL-4 or IL-17A and/or OSM in 2% FBS/RPMI or serum-free RPMI. 24-hour supernatants were assessed by ELISA for Eotaxin-1/CCL11. **P<0.05*; ****P<0.0001* compared to control; *# p<0.05* compared to either cytokine alone (One-Way ANOVA) with Bonferroni’s post-test. **Figure S5.** HASMC cell signaling in serum-free conditions. HASMC cultures from two different subjects were stimulated as indicated (10 ng/ml) in serum-free RPMI/1% BSA for 20 minutes and lysed. Total cell lysates were assessed for the indicated proteins by immunoblot analysis. **Figure S6.** Time course of HASMC responses. HASMC cultures were stimulated with 10 ng/ml of the indicated cytokines in 2% FBS/ RPMI (Left panel) or serum-free RPMI/1% BSA (Right panel) for 12, 24 and 48 hours. Cell supernatants were assessed for MCP-1 (A), IL-6 (B) and Eotaxin-1/CCL11 (C).

## Abbreviations

ELISA: Enzyme-linked immunosorbent assay
FBS: Fetal bovine serum
gp130: Glycoprotein 130
HASMC: Human airway smooth muscle cells
MAPK: Mitogen-activated protein kinase
OSM: Oncostatin M
PBS: Phosphate-buffered saline
PDAR: Pre-determined assay reagents
STAT: Signal transducers and activators of transcription
TNF: Tumor necrosis factor
VEGF: Vascular endothelial growth factor
