# Supplementary material for: Regulation of IL-17A responses in human airway smooth muscle cells by Oncostatin M
Source: Respir Res. 2015 Feb 7;16(1):14. doi: 10.1186/s12931-014-0164-4 (PMC4332894; doi:10.1186/s12931-014-0164-4)
Supplement: Additional file 1: Figure S1. — HASMC responses to gp130 cytokines. HASMC cultures were stimulated with 10ng/ml of LIF, IL-6, IL-31 or IL-11 and with IL-17A (0–10 ng/ml). 24-hour supernatant levels were quantified by ELISA for MCP-1/CCL-2 (upper panel) or IL-6 (lower panel). Figure S2. mRNA levels of Eotaxin-3. HASMC cultures were assessed for eotaxin-3 RNA (qRT-PCR) after 6 hours (top) and 18 hours (bottom) of stimulation with the indicated cytokines at 5ng/ml (OSM at 2 ng/ml). Fold change from control, corrected to β-Actin, is shown. Figure S3. IL-8 protein regulation by OSM/IL-17A. HASMC cultures were stimulated with 5 ng/ml of IL-4, IL-13 or IL-17A and OSM (1 or 5 ng/ml). 18-hour supernatant levels were quantified by ELISA. ***P<0.0001 (One-Way ANOVA) compared to control or OSM treatment alone. Figure S4. OSM stimulation in serum free conditions. HASMC cultures were stimulated in serum-free RPMI/1% BSA. 24-hour supernatant levels were quantified by ELISA for (A) MCP-1 and (B) IL-6. Panel C: HASMC cultures were stimulated with 10 ng/ml of IL-4 or IL-17A and/or OSM in 2% FBS/RPMI or serum-free RPMI. 24-hour supernatants were assessed by ELISA for Eotaxin-1/CCL11. *P<0.05; ***P<0.0001 compared to control; # p<0.05 compared to either cytokine alone (One-Way ANOVA) with Bonferroni’s post-test. Figure S5. HASMC cell signaling in serum-free conditions. HASMC cultures from two different subjects were stimulated as indicated (10 ng/ml) in serum-free RPMI/1% BSA for 20 minutes and lysed. Total cell lysates were assessed for the indicated proteins by immunoblot analysis. Figure S6. Time course of HASMC responses. HASMC cultures were stimulated with 10 ng/ml of the indicated cytokines in 2% FBS/ RPMI (Left panel) or serum-free RPMI/1% BSA (Right panel) for 12, 24 and 48 hours. Cell supernatants were assessed for MCP-1 (A), IL-6 (B) and Eotaxin-1/CCL11 (C). [file 12931_2014_164_MOESM1_ESM.pdf]

Supplementary figure 1

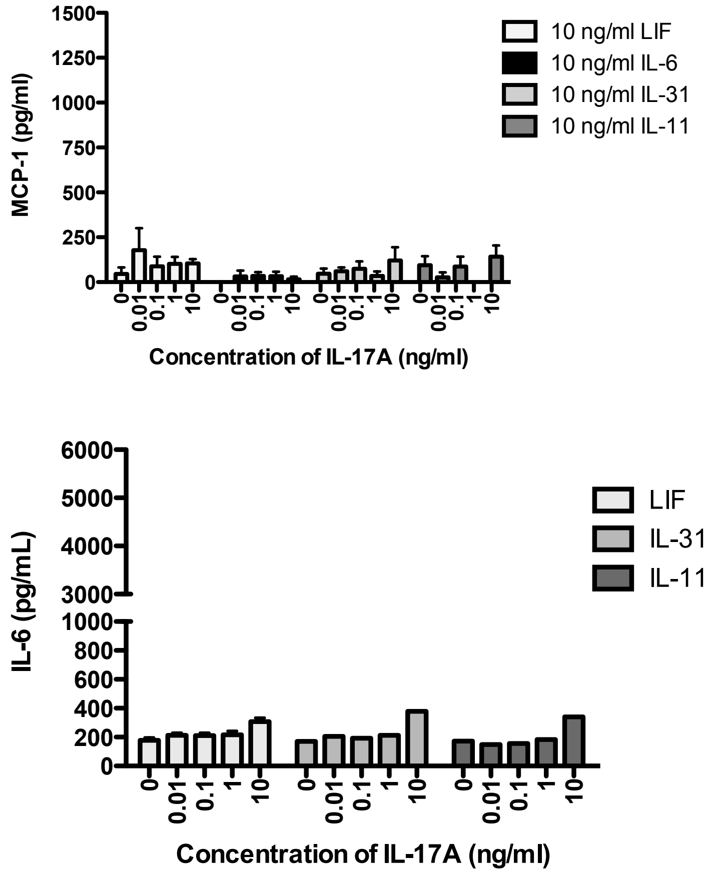

Supplemental Figure 1: HASMC response to LIF, IL-6, IL-31 and IL-11.HASMC cultures were prepared and stimulated as in Fig 1 with 10ng/ml of LIF, IL-6, IL-31 or IL-11 and concentrations of IL-17A up to 10 ng/ml. 24- hour supernatants were collected and cytokine concentrations were quantified by ELISA for MCP-1/CCL-2 (upper panel) or IL-6 (lower panel).

Supplementary figure 2

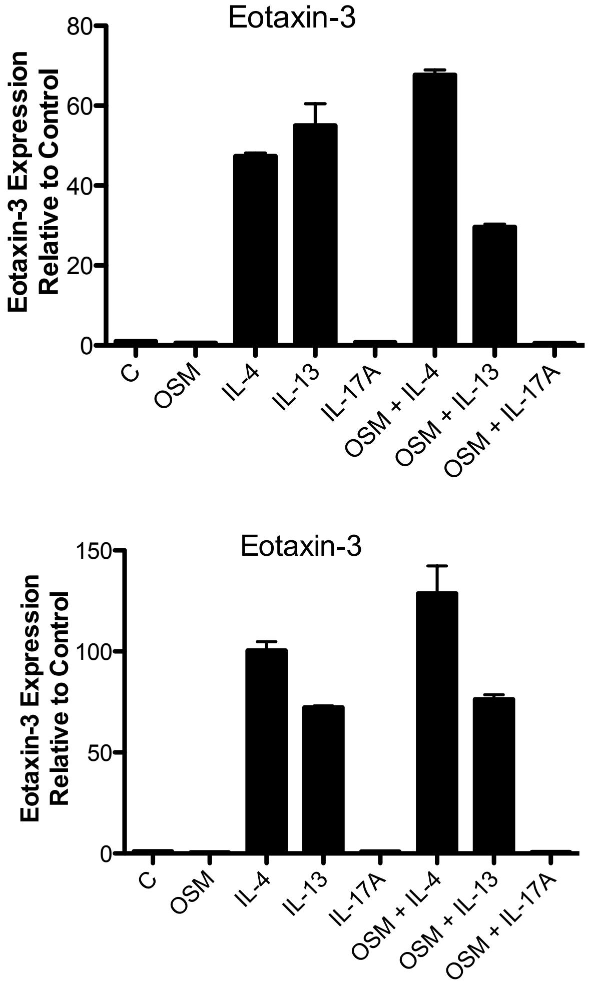

Supplemental Figure 2: Steady state mRNA levels of Eotaxin-3 .HASMC were plated and prepared for RNA analysis as in methods after 6 hours(top) and 18 Hours (Bottom) of stimulation with the indicated cytokines at 5ng/ml (OSM at 1 ng/ml). RNA was prepared and analyzed by qRT-PCR using probes for eotaxin-3 as indicated. Levels are expressed as fold change relative to control using b-actin as a reference control gene.

Supplementary Figure 3

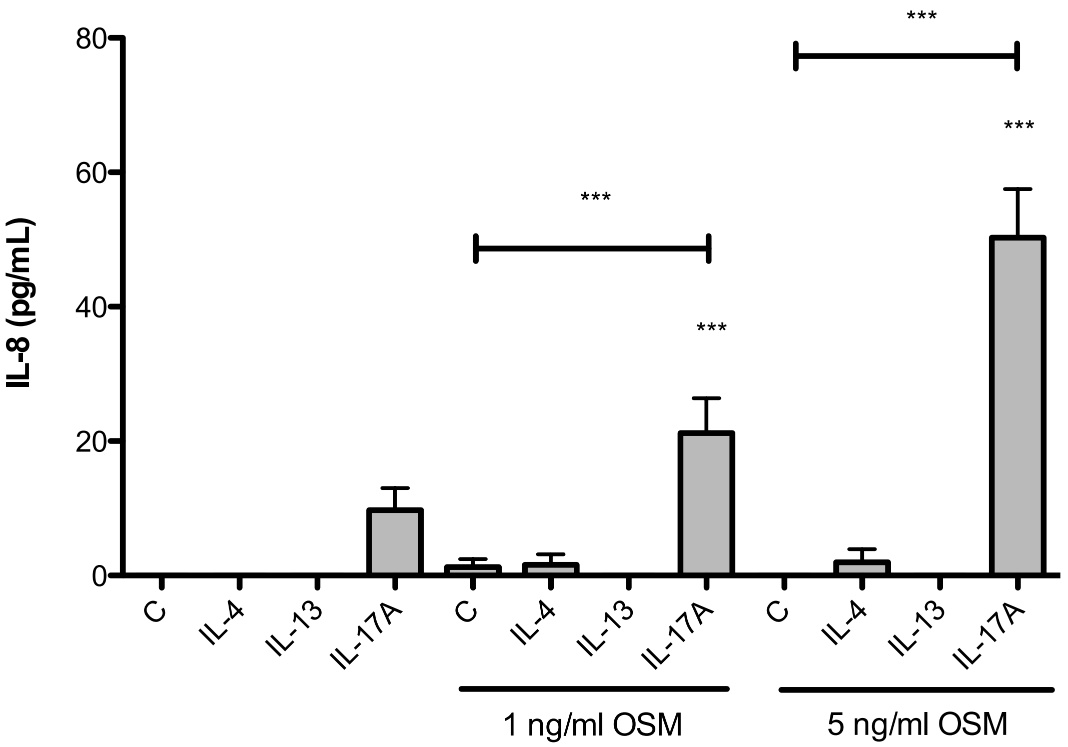

Supplemental Figure 3: IL-8 protein regulation by OSM/IL-17A from HASMC. HASMC cultures were prepared and stimulated as in Fig 1 with 5 ng/ml of IL-4, IL-13 or IL-17A and concentrations of OSM at 0.5, 1 and 5 ng/ml. 18- hour supernatants were collected and cytokine concentrations were quantified by ELISA for IL-8/ CXCL8. ***=P<0.0001 using One-Way ANOVA comparing treatment to unstimulated control (C). *** (above bars) indicate P<0.0001 comparing treatment to OSM alone at the indicated concentration.

Supplementary Figure 4

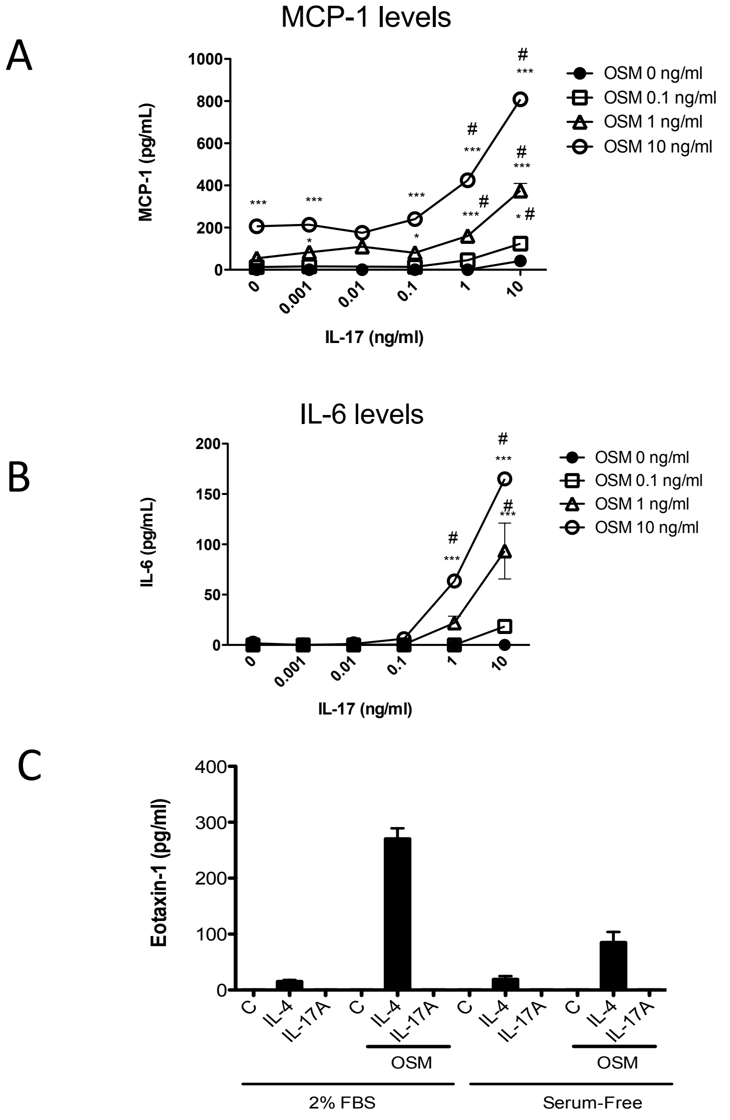

Supplemental Figure 4: OSM synergistic in serum free conditions. Panel A-B: HASMC cultures were prepared and stimulated as in Fig 2A with 0-10 ng/ml IL-17A and concentrations of OSM at 0.1, 1 and 10 ng/ml in serum free RPMI containing 1% BSA. 24- hour supernatants were collected and cytokine concentrations were quantified by ELISA for(A) MCP-1 and (B) IL-6. Panel C: HASMC cultures were prepared and stimulated as described previously with 10 ng/ml of IL-4 or IL-17A and/or OSM in 2%FBS/RPMI or serum free RPMI 24-Hour supernatants were assessed by ELISA for Eotaxin-1/CCL11. *=P<0.05; ***=P<0.0001; # =p<0.05 comparing treatment to either cytokine alone using two way ANOVA with Tukey post-test

Supplementary Figure 5

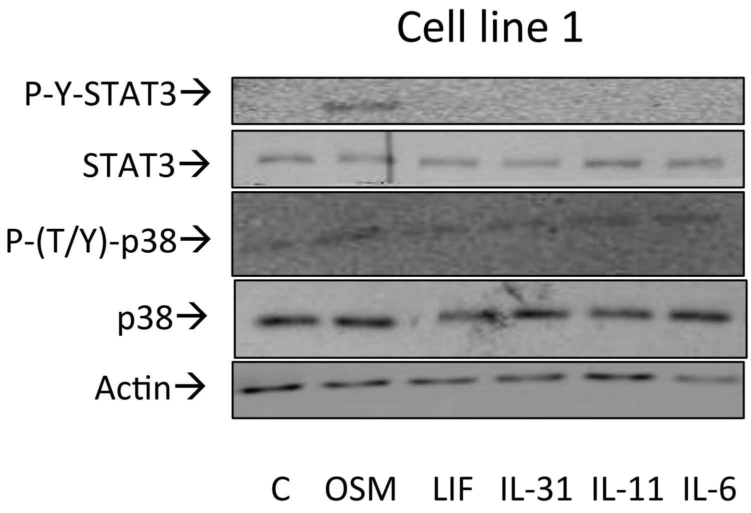

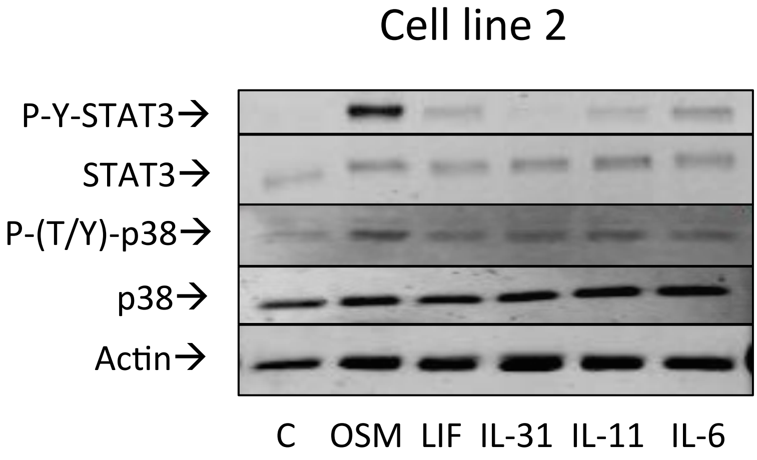

Supplemental Figure 5: HASMC cell signaling in response to OSM and gp130 cytokines in serum-free conditions. HASMC cultures from two different subjects were prepared as previously described and stimulated with 10 ng/ml of OSM, LIF, IL-31, IL-11 or IL-6 in serum-free RPMI medium containing 1% BSA for 20 minutes and lysed. Total cell lysates were probed for indicated proteins using standard immunoblot procedures

Supplementary Figure 6

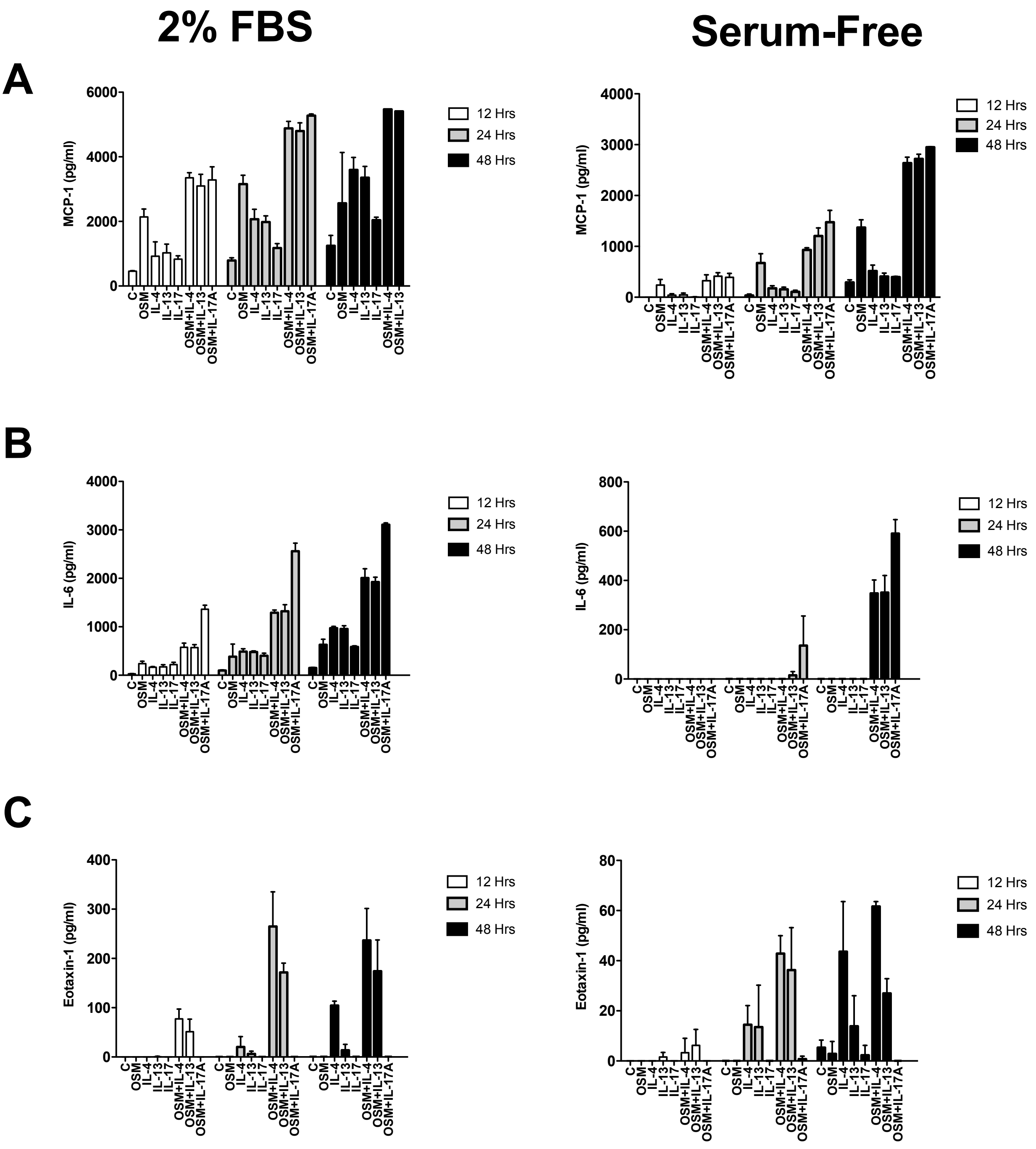

Supplemental Figure 6: Time course of HASMC responses to OSM in combination with IL-4, IL-13 or IL-17A in serum free and serum containing conditions. HASMC cultures were prepared as described previously and stimulated with 10 ng/ml of IL-4, IL-13 or IL-17A with or without 10 ng/ml of OSM in 2% FBS RPMI(Left panel) or serum free RPMI containing 1% BSA (Right panel) for 12 (white bars), 24 (grey bars) and 48 hours (black bars). Cell supernatants for each time point were assessed for protein levels of MCP-1 (A), IL-6 (B) and Eotaxin-1/CCL11 (C) by ELISA.
